# Involvement of HCN Channel in Muscarinic Inhibitory Action on Tonic Firing of Dorsolateral Striatal Cholinergic Interneurons

**DOI:** 10.3389/fncel.2016.00071

**Published:** 2016-03-22

**Authors:** Zhe Zhao, Kang Zhang, Xiaoyan Liu, Haitao Yan, Xiaoyun Ma, Shuzhuo Zhang, Jianquan Zheng, Liyun Wang, Xiaoli Wei

**Affiliations:** State Key Laboratory of Toxicology and Medical Countermeasures, Department of Biochemical Pharmacology, Beijing Institute of Pharmacology and ToxicologyBeijing, China

**Keywords:** cholinergic interneurons, muscarinic receptor, HCN channel, scRT-PCR, muscarinic inhibitory

## Abstract

The striatum is the most prominent nucleus in the basal ganglia and plays an important role in motor movement regulation. The cholinergic interneurons (ChIs) in striatum are involved in the motion regulation by releasing acetylcholine (ACh) and modulating the output of striatal projection neurons. Here, we report that muscarinic ACh receptor (M receptor) agonists, ACh and Oxotremorine (OXO-M), decreased the firing frequency of ChIs by blocking the hyperpolarization-activated cyclic nucleotide-gated (HCN) channels. Scopolamine (SCO), a nonselective antagonist of M receptors, abolished the inhibition. OXO-M exerted its function by activating the G_i/o_ cAMP signaling cascade. The single-cell reverse transcription polymerase chain reaction (scRT-PCR) revealed that all the five subtypes of M receptors and four subtypes of HCN channels were expressed on ChIs. Among them, M2 receptors and HCN2 channels were the most dominant ones and expressed in every single studied cholinergic interneuron (ChI).Our results suggest that ACh regulates not only the output of striatal projection neurons, but also the firing activity of ChIs themselves by activating presynaptic M receptors in the dorsal striatum. The activation of M2 receptors and blockage of HCN2 channels may play an important role in ACh inhibition on the excitability of ChIs. This finding adds a new G-protein coupled receptor mediated regulation on ChIs and provides a cellular mechanism for control of cholinergic activity and ACh release in the dorsal striatum.

## Introduction

As a prominent nucleus in the basal ganglia, striatum serves as a center of input and integration for cortical, thalamic, and midbrain afferents. The striatum is functionally divided into two parts, along a dorsolateral/ventromedial axis, which exert different roles in cognitive, affective, and limbic functions (Smith and Kieval, 2000; Voorn et al., 2004). The striatum is composed of projection neurons, cholinergic interneurons (ChIs), and other GABAergic interneurons (Vincent et al., 1983; Chesselet and Graybiel, 1986; Smith and Parent, 1986; Cowan et al., 1990; Bennett and Bolam, 1993).

ChIs only take a small fraction of striatal neurons (1–3%), but have widespread connections throughout the striatum (Kawaguchi et al., 1995; Tepper and Bolam, 2004). They synthesize, transport, and secrete acetylcholine (ACh; Woolf and Butcher, 1981; Wang et al., 2006; Ding et al., 2010; Goldberg et al., 2012). Despite their small numbers, these giant and spiny ChIs are responsible for striatal levels of ACh, which is among the highest in the brain (Mesulam et al., 1992; Contant et al., 1996). Increased release of ACh by ChIs has been shown to contribute to structural changes and distorted network function in the striatum (Pisani et al., 2007).

ChIs have been proposed to regulate the duration, strength, and spatial pattern of action potentials in striatal local circuits (Galarraga et al., 1999; Calabresi et al., 2000; Koós and Tepper, 2002). Their dysfunctions are involved in behavior and other movement disorders such as Parkinson’s disease (Apicella et al., 1997; Blazquez et al., 2002; Morris et al., 2004; Joshua et al., 2008; Witten et al., 2010). By activating muscarinic receptors (M receptors), ACh exerts its profound modulatory effect on postsynaptic neurons. M receptors are divided into two classes: M1-class (M1, M3, and M5) and M2-class (M2, M4). M1-class receptors couple to G_αq_ proteins that activate phospholipase C (PLC) signal cascade. M2-class receptors preferentially couple to G_αi_ proteins that inhibit adenylyl cyclase (AC) and downregulate intracellular cAMP content. M receptors have been reported to be widely expressed in the striatum, and all five M receptor subtypes (M1–5) are expressed in the dorsal striatum (Eglen, 2012). M1-class receptors are mostly distributed on the postsynaptic membrane, while M4 receptors are restricted to the striatonigral medium-size spiny neurons (MSNs) and neuropeptide-Y releasing interneurons (Ince et al., 1997; Yan et al., 2001). M2 receptors are considered to be mainly expressed in ChIs (Weiner et al., 1990; Bernard et al., 1992) where they function as cholinergic autoreceptors and regulate ACh release (Alcantara et al., 2001). However, M4 receptors are also reported to be expressed on ChIs as autoreceptors (Bernard et al., 1992; Hersch et al., 1994; Yan and Surmeier, 1996).

ChIs are pacemaking neurons and present distinct burst-pause patterns in their tonic firings during motor learning and reward-related behaviors. Autonomous pacemaking activity in ChIs is mainly driven by the hyperpolarization-activated cyclic nucleotide-gated (HCN) channels (Bennett and Wilson, 1999; Wilson, 2005). The HCN currents (I_h_) exert regulatory effects on intrinsic ChIs’ excitability by depolarizing the membrane to its firing threshold of action potentials (Bennett et al., 2000; Wilson, 2005).

In mammals, the HCN channels are encoded by four genes (HCN1-4), which present various electrophysiological properties, activation kinetics, and cAMP-sensitivity, and are widely expressed throughout the heart and the central nervous system (Santoro et al., 2000; Ulens and Siegelbaum, 2003; Biel et al., 2009). I_h_ is considered to be involved in at least four physiological processes: “(1) control of pacemaker activity; (2) control and limitation of resting potential; (3) control of membrane resistance and dendritic integration; and (4) regulation of synaptic transmission” (Robinson and Siegelbaum, 2003). All four HCN isoforms are expressed in the brain (Biel et al., 2009). HCN expression patterns have been characterized in some nucleus, such as hippocampus. However, the identification of HCN channel subunits expressed in ChIs is unclear.

It has been confirmed that dopamine modulates the pause response in tonic firing in ChIs by inhibiting HCN channels (Deng et al., 2007). And there are also reports that ACh can regulate the ChIs’ activity through voltage- and/or ligand-gated channels (Yan and Surmeier, 1996; Calabresi et al., 1998; Pisani et al., 1999; Ding et al., 2006, 2010; Bonsi et al., 2008). However, whether HCN channels are directly involved in the muscarinic modulation on the excitability of ChIs has not been reported. We speculate that application of M receptor agonist could activate M receptors expressed on ChIs and thus downregulate internal cAMP, which would result in the reduction of I_h_ and inhibition of spontaneous firing.

To test our hypothesis, we first used single cell RT-PCR with subtype-specific primers to identify the distribution of HCN and M receptor subtypes. Then, we observed the effect of M receptor agonists on I_h_ and firing activity of ChIs. Our data reveal that all the HCN subunits are expressed on ChIs but HCN2 is the most abundant one. In terms of M receptors, all four subtypes are found in some ChIs but M2 is predominantly expressed. Furthermore, application of M receptor agonist depresses the firing activity of ChIs. Therefore, we propose that the activation of M2 receptors and blockage of HCN2 channels underlie the ACh inhibition on the excitability of ChIs.

## Materials and Methods

### Brain Slice Preparation

All experiments were approved by the Animal Research Advisory Committee of Beijing Institute of Biological Science and in accordance with the NIH guideline (Publication No. 85–23, revised 1985) to the care and use of laboratory animals. Preparation of striatal slices was carried out similar to those previously described (Bennett and Wilson, 1999; Deng et al., 2005; Sciamanna et al., 2011). Briefly, male Sprague Dawley rats (14–16 days old) were killed by cervical dislocation. The brain was quickly removed from the skull and submerged in ice-cold (4°C) oxygenated sucrose solution containing (in mM): 230 sucrose, 2.5 KCl, 1.25 NaH_2_PO_4_, 24 NaHCO_3_, 10 glucose, 10 MgSO_4_, 0.5 CaCl_2_, 2 sodium pyrurate, and adjusted pH 7.4 with NaOH, 295–305 mOsm/L. Coronal striatal slices (300 μm) were cut using a vibratome (MA752, Campden instruments). Slices, put in a chamber (Warner instruments) bubbled with a 95% O_2_ and 5% CO_2_ gas mixture, were incubated in the standard NaHCO_3_-buffered saline solution containing (in mM): 126 NaCl, 2.5 KCl, 1.25 NaH_2_PO_4_, 26 NaHCO_3_, 10 glucose, 2 sodium pyrurate, 2 CaCl_2_, 2 MgCl_2_, pH 7.4 with HCl (300–305 mOsm/l), for 30 min at 32°C. The chamber was then maintained at room temperature continuously bubbled with O_2_/CO_2_ gas mixture. The slices could be stored *in vitro* for several hours while maintaining excellent viability prior to electrophysiological experiment.

### Electrophysiological Recording

A single slice was transferred to the recording chamber and submerged in a continuously flowing NaHCO_3_-buffered saline (1.5–2 ml/min) bubbled with a 95% O_2_ and 5% CO_2_ gas mixture at room temperature (~25°C). Recording electrodes were prepared from borosilicate glass (Sutter instruments, Novato, CA, USA) using a horizontal electrode puller (P-97, Sutter instruments, Novato, CA, USA). The electrodes had resistance of 2–4 MΩ when filled with the internal solution consisted of (in mM): 130 K^+^-gluconate, 10 HEPES, 10 KCl, 5 EGTA, 1 CaCl_2_, 1 MgCl_2_, 2 Na_2_ATP, 0.5 Na_3_GTP, pH 7.4, 295–300 mOsm/L. The slice was visualized with a 40× water-immersion objective (NIR Apo, Nikon, Japan) using standard infrared and differential interference contrast (IR-DIC) microscopy and a CCD camera. Cells in the dorsolateral striatum up to ~50 μm beneath the slice surface were patched and monitored. Recording in normal current-clamp or voltage-clamp mode was performed with an Axon 200B amplifier (Molecular devices, Foster city, CA, USA) and Clampex 10.1 software (Molecular devices) at room temperature (~25°C; Bennett and Wilson, 1999; Nolan et al., 2003; Hawkins et al., 2015). After tight-seal (>1 GΩ) formation, fast and slow capacitance compensation was performed. During the whole-cell recording, series resistance was compensated (80–90%) and monitored periodically. Neurons were excluded from the analysis when their series resistance was above 50 MΩ or changed by more than 25% during the experiment. Data were filtered at 2 kHz and acquired at sampling rate of 10 kHz.

Modified NaHCO_3_-buffered saline for recording I_h_ had the composition (mM): 115 NaCl, 5 KCl, 1.25 NaH_2_PO_4_, 25 NaHCO_3_, 10 glucose, 2 sodium pyrurate, 2 CaCl_2_, 2 MgCl_2_, pH 7.4. BaCl_2_ (1 mM) and TTX (0.5 μM) were added to the saline to block inward rectifier K^+^ and Na^+^ channels, respectively. CdCl_2_ (0.1 mM), 4-aminopyridine (2 mM), and tetraethylammonium (5 mM) were also added to saline to block voltage-dependent Ca^2+^ and K^+^ channels, respectively (Nolan et al., 2003; Deng et al., 2007). In the present study, synaptic blockers were not used because the ChIs receive minimal synaptic inputs *in vitro*, and these inputs have an undetectable effect on the spontaneous firing rates and patterns exhibited by these cells (Bennett and Wilson, 1999). I_h_ was activated using hyperpolarizing voltage steps from a holding potential of −50 mV to −140 mV in 10 mV decrements in voltage-clamp model. To ensure the stability of whole-cell recordings, the sweep start-to-start interval was 5 s. The amplitude of I_h_ was calculated by subtracting the current value at the onset of hyperpolarizing voltage (peak amplitude) from that at the end (mean current of the 20 ms before the termination of each voltage step; Kodirov et al., 2014).

### Histochemical Staining for Biocytin-Loaded Slices

To identify the morphology of recorded cells, 0.2% (w/v) biocytin was added into the pipette solution to stain cells by diffusion (Horikawa and Armstrong, 1988). After termination of recording, the slice containing the cell injected with biocytin was fixed by immersion in 4% paraformaldehyde in 0.1 M phosphate buffer, pH 7.4 at 4°C for 10 h and then incubated in PBS containing 1% Triton x-100 (TX) for 2 h at room temperature. The slices were then washed with 0.01 M PBS three times for 10 min each time. And then the slices were incubated in PBS containing 3% H_2_O_2_ for 10 min to suppress endogenous peroxidase activity and washed in PBS three times for total of 15 min. They were then incubated in PBS containing avidin-biotin-peroxidase complex (ABC solution, Fuzhou Maixin company, China) for 10 min. 0.05% (w/v) 3,3′-Diaminobenzidine tetrahydrochloride (DAB) 0.1–0.2 ml was dropped on the slices to react for 3–5 min, then washed in PBS three times for 5 min each.

To ensure the biocytin-loaded cells were ChIs, the biocytin-loaded slices were further processed with immunofluorescence histochemical staining. The slices were blocked using rabbit serum at room temperature for 1 h and then incubated with a goat polyclonal antibody against choline acetyltransferase (ChAT; Millipore, Cat# AB144P, RRID: AB_2079751 using at 1:100) in PBS overnight at 4°C. After 3 × 5 min washing in PBS, the slices were incubated in rabbit serum containing the Rhodamine (TRITC)-conjugated affinipure rabbit anti-goat IgG (1:200) for 1 h at room temperature. After 3 × 5 min washing in PBS once more, the slices were glycerol-mounted on slides and photographed under a fluorescence microscope (BX51 Olympus optical, Japan).

### Histochemical Staining for Perfusion-Fixed Brains

For histochemical experiments, animals of similar age to those used for electrophysiological experiments were used. The animals were deeply anesthetized with overdose of Nembutal, and perfused transcardially with phosphate buffered saline first, followed by 200 ml ice-cold fixative containing 4% paraformaldehyde in PBS, pH7.4. The brain was removed carefully, cut sagittally at the midline and post-fixed in the same fixative for 3 h at 4°C. After incubation in PBS containing 30% (w/v) sucrose for 3 d at 4°C, the brain was sectioned at 12 μm thickness, and slices containing the striatum were mounted on polylysine-coated slides. Four primary antibodies were ordered from commercial companies: a rabbit anti-HCN 1 antibody (Alomone Labs, Cat# APC-056, RRID: AB_2039900 using at 1:100), a rabbit anti-HCN 2 antibody (Alomone Labs, Cat# APC-030, RRID: AB_2313726 using at 1:100), a rabbit anti-M2 receptor antibody (Abcam, Cat# ab109226, RRID: AB_10858602 using at 1:200), and a mouse anti-M4 receptor antibody (Abcam, Cat# ab77956, RRID: AB_1566454 using at 1:200).

Sections were permeablized in 0.1% TX in PBS for 1 h, blocked with normal serum, which was from the same host of secondary antibody, for 1 h, and incubated overnight at 4°C with the primary antibody diluted in PBS containing 1% BSA. Then, sections were rinsed with PBS for 3 × 5 min and transferred to secondary antibody in blocking serum for 1 h at room temperature in dark. After another three washes, the sections were mounted in medium fluoroshield^TM^ with DAPI (Sigma-aldrich F6057, Saint Louis, MO, USA) and coverslipped. Then they were subsequently photographed using a confocal laser scanning microscope (Zeiss, LSM510). The control experiment for immunohistochemical labeling specificity of the secondary antibody includes the omission of the primary antibody and the use of preimmune normal serum (Tozzi et al., 2011). The result indicated that there was no nonspecific labeling of neural soma or processes.

### Single-Cell Reverse Transcription-Polymerase Chain Reaction (scRT-PCR)

HCN channel and M receptor mRNAs in striatal ChIs were detected by using techniques similar to those described previously (Surmeier et al., 1996; Yan and Surmeier, 1996; Tkatch et al., 2000). Neurons were subjected to whole cell voltage-clamp recording before aspiration. To maximize mRNA yields, some neurons were aspirated without recording with an electrode containing ~4 μl of sterile water. Neurons were aspirated into the patch electrode by applying negative pressure. After aspiration, the electrode was removed from the holder, the tip was broken, and the content was ejected into a 0.5 ml eppendorf tube containing 0.5 μl of oligo-dT, 0.5 μl of random primer, 0.25 μl of RNasin (40U/μl). The primer mixture was heated to 70°C for 5 min and then immediately chilled in ice water for at least 5 min. The reverse transcription (RT) reaction mixture, total of approximately ~20 μl, was composed of the pipette tip content, primer mixture, GoScript^TM^ 5× Reaction buffer (4 μl), MgCl_2_ (2.4 μl), dNTP (1 μl), RNasin (0.25 μl), GoScript^TM^ Reverse Transcriptase (1.0 μl), and nuclease-free water (6.35 μl). Single-strand cDNA was synthesized following this step: annealing at 25°C for 5 min, extending at 42°C for 60 min, inactivating reverse transcriptase at 70°C for 15 min, and then icing. The RNA strand in the RNA-DNA hybrid was removed by adding 1 μl of RNase H (2 U/μl) and incubating for 20 min at 37°C. All reagents were obtained from Promega Inc (Madison, WI, USA). All the semi-quantitative experiments presented here were conducted with the same enzyme lot.

The cDNA from the RT in a single striatal neuron was amplified using PCR protocols modified from Surmeier’s report (Surmeier et al., 1996). Amplification was performed with one of the two protocols. The conventional one-stage amplification protocol was carried out in a thermal cycler (Applied Biosystems) with thin-walled plastic tubes. Detection experiments were carried out using one-tenth of the single-cell cDNA (2μl) as a template for the PCR. Reaction mixture contained 2–2.5 mM MgCl_2_, 0.2 mM each dNTP, 0.8 μM primers, 1.25U GoTaq G2 flexi DNA polymerase, and 10 μl green GoTaq flexi buffer. Nuclease-free water was added to final volume of 50 μl. The thermal cycling program for all reactions was set in three steps: step1, 94°C for 3 min; step2, for 42 cycles, 94°C for 1 min, 58°C for 1 min, 72°C for 1 min; and step 3, 72°C for 5 min.

In the semi-quantitative RT-PCR experiment, the modified two-stage amplification protocol was designed to maximize our ability to detect low abundance of mRNAs of HCN channels or M receptors (Yan and Surmeier, 1996). In the first stage, 2μl cDNA from RT was used as template (Surmeier et al., 1996; Yan and Surmeier, 1996; Tkatch et al., 2000). All HCN channel or M receptor primers were added to a reaction mixture containing the same concentration of reagents as in the conventional one-stage amplification protocol, except for slightly elevated MgCl_2_ (3.5~4.0 mM) and dNTPs (1.0 mM; Chamberlain and Chamberlain, 1994). The same program as the conventional one-stage amplification protocol was performed in 13 cycles. In the second stage, an aliquot (1/10) of the first stage PCR product (5μl) was serially diluted and used as a template for a second round of “touch down” PCR amplification with each pair of specific primers. Thirty two cycles were performed with the same program as the first stage.

The PCR primers were synthesized either by the Beijing Bomaide or Beijing Huada Inc. PCR primers for glutamate decarboxylase 67 (GAD67), β-actin, GAPDH, ChAT, HCN channels, and M receptors were described previously (Yan and Surmeier, 1996; Tkatch et al., 1998; Budde et al., 2005). PCR procedures were performed using procedures designed to minimize the chance of cross-contamination (Cimino et al., 1990). Negative controls for contamination from extraneous and genomic DNAs were run for every batch of neurons. To ensure that genomic DNA did not contribute to the PCR products, neurons were aspirated and processed in the normal manner, except that the reverse transcriptase was omitted. Contamination from extraneous DNA was checked by replacing the cellular template with water. Both controls were consistently negative in these experiments.

PCR products were visualized by staining with ethidium bromide and analyzed by the electrophoresis in 2% agarose gels. In representative cases, amplicons were purified from the gel, and products were sequenced and verified in Beijing Bomaide or Beijing Huada Inc.

### Drug Application and Data Analysis

All drugs were purchased from Sigma-Aldrich (St. Louis, MI, USA) except noted specially. Drugs were dissolved as concentrated stocks in either water or DMSO and stored at −20°C. When DMSO stock solution was used, equivalent amounts of DMSO were added to buffer as controls, and the final concentration of DMSO should not exceed 0.1%. Working solutions with different drugs were prepared just before use. During experiments, drugs, except otherwise marked, were applied in the flowing bath solutions. GDP-β-S was added into the intracellular solution in recording pipette. Total replacement of the medium in the recording chamber occurred within 1 min.

Data analysis was performed with software including Clampfit Version 10.2, Prism Version 6.0, and Origin Version 9.0. The relative I_h_, as shown in Figures 2, 7, was determined as B/A*100%, in which A and B represents the I_h_ recorded before and after the application of drugs, at the −140 mV hyperpolarizing voltage, separately. All results were presented as mean ± SD. Statistical analysis was performed using student’s *t*-test (paired where relevant), the one-way ANOVA, and the two-way ANOVA. Difference of *p* < 0.05 was considered statistically significant. The threshold probability of single cell PCR detection was fitted by the Gaussian curve regression.

## Results

### Identification of ChIs in Dorsolateral Striatum

**Figure 1:**
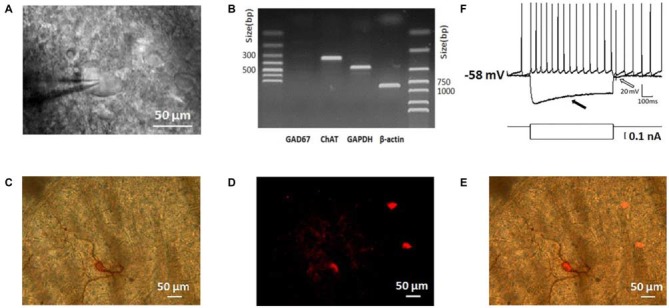
**Morphological, physiological, electrophysiological, and immunohistochemical staining characterization of striatal cholinergic interneurons (ChIs). (A)** An IR-DIC image of a dorsolateral striatal slice illustrating the characteristic appearance of giant interneurons. **(B)** ChAT was shown in single-cell reverse transcription polymerase chain reaction (scRT-PCR) experiment product. The absence of GAD 67 indicated aspirated cellular content wasn’t contaminated by MSNs. **(C)** Micrograph of a giant cell loaded with biocytin and subsequently stained immunohistochemically. **(D)** Immunofluorescence staining of ChIs in biocytin-loaded slices. **(E)** Merge of **(C)** and **(D)**. The targeted cell intracellular loaded biocytin was co-stained by ChAT, inferring the giant aspiny ChIs. **(F)**, Depolarizing somatic current injection elicited a train of regular spiking followed by a large hyperpolarization (indicated by a hollow arrow). Negative current injection caused a large hyperpolarization followed by a sag (indicated by a filled arrow).

To guarantee the neurons we studied are ChIs, we identified them based on their morphological, electrophysiological, and histochemical features (Paxinos and Watson, 1986; Kawaguchi, 1993; Bennett and Wilson, 1998). As shown in Figure 1A, neurons under IR-DIC visualization with large soma and thick primary dendrites were initially targeted. In scRT-PCR experiments, the cells selected preferentially in morphology transcribed ChAT mRNA while GAD67 products were not detected (Figure 1B), which could exclude the contamination of MSNs. Examination of biocytin-filled neurons revealed that the thick primary dendrites branched further to form secondary and higher order smaller-diameter dendrites (Figure 1C). In several cases, the identity of the recording neuron was further verified through a histochemical staining (Figure 1D). Every neuron loaded with biocytin also expressed ChAT (Figure 1E).

Depolarization of the membrane potential elicited repetitive firings in recorded neurons that were followed by a large-amplitude and long duration of after-hyperpolarization (AHP; indicated by a hollow arrow in Figure 1F). On the other hand, an initial hyperpolarization was followed by a subsequent sag (indicated by a solid arrow in Figure 1F), indicating the presence of a cation current, I_h_ presumably. In slices, the majority of neurons was tonically active and showed spontaneous activity. Moreover, the recorded neurons present electrophysiological characters that are consistent with that of the striatal ChIs described previously (Bennett and Wilson, 1999; Sanchez et al., 2011; Ponterio et al., 2013).

### Characterization of I_h_ in ChIs

**Figure 2:**
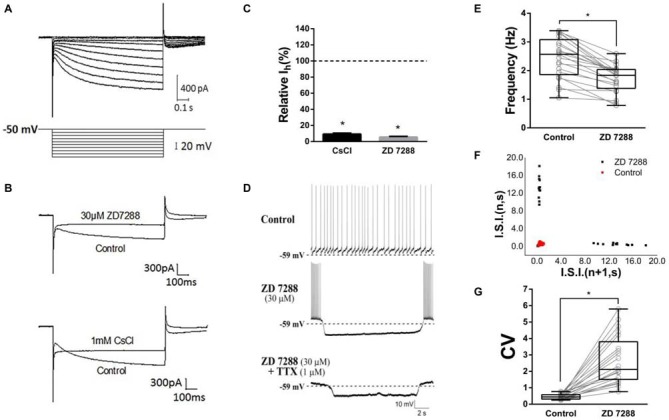
**Contribution of HCN currents (I_h_) to tonic firing. (A)** The specific I_h_ was isolated in dorsolateral striatal ChIs. **(B)** Example traces of Current evoked at the −140 mV hyperpolarizing voltage before and after bath application of hyperpolarization-activated cyclic nucleotide-gated (HCN) channel blockers, ZD7288 (30 μM) or Cs^+^ (1 mM). **(C)** ZD7288 and CsCl significantly blocked the I_h_. **(D)** ZD7288 (30 μM) induced a discontinuously hyperpolarized pause and altered the spiking pattern. The induced action potential bursts were block by TTX but not the subthreshold membrane potential oscillation. **(E)** ZD7288 significantly decreased the overall event frequency of spiking (*p < 0.001, paired student’s *t*-test, *n* = 21). **(F)** A representative scatter of joint consecutive interspike interval (I.S.I.) plot of the discharge. **(G)** ZD7288 significantly increased the coefficient of variation (CV) of the I.S.I frequency (*p < 0.001, paired student’s *t*-test, *n* = 21).

As Deng et al. (2007) reported, we isolated I_h_ from ChIs with the modified NaHCO_3_-buffered saline described in methods section. Time- and voltage- dependent inward currents were activated in a voltage-clamp mode by a series of hyperpolarizing pulses from −50 mV to −140 mV in a 10 mV decrement (Figure 2A). To confirm the inward currents are I_h_, HCN channel blocker ZD7288 and Cs^+^ were used. When ZD7288 or Cs^+^ were applied to the perfusion, the hyperpolarized sag currents were remarkably depressed (Figure 2B), which confirmed the I_h_ identification. As shown in Figure 2C, at the −140 mV hyperpolarizing voltage, application of ZD7288 (30 μM) dramatically blocked the I_h_ by 94.50% ± 0.99% (*n* = 7), and the presence of CsCl (1 mM) inhibited the I_h_ by 90.74% ± 1.13% (*n* = 5) respectively.

To test whether I_h_ plays a role in determining the firing pattern of ChIs, the effect of ZD7288 was observed in standard NaHCO_3_-buffered saline. We found that application of ZD7288 (30 μM) induced a discontinuously hyperpolarized pause and changed the spiking pattern into oscillation with intra-bursting frequency of 6.21 ± 2.18 Hz (middle panel in Figure 2D, *n* = 21). Application of TTX only abolished the burst spiking but did not change the effects of subthreshold membrane potential oscillation (lower panel in Figure 2D, *n* = 21). In presence of ZD7288, the overall event frequency of spiking was reduced from 2.44 ± 0.71 Hz to 1.72 ± 0.48 Hz (Figure 2E, *n* = 21, *p* < 0.001). The coefficient of variation (CV) of joint consecutive inter-spike interval (I.S.I.) was used to describe the irregularity of pacemaking rhythm in ChIs. Application of ZD7288 altered CV value significantly from 0.46 ± 0.16 of the control group to 2.67 ± 1.46 (Figures 2F,G, *n* = 21, *p* < 0.001). These results indicated that I_h_ is critical to the firing pattern of ChIs.

### HCN Channels Expression on ChIs

**Figure 3:**
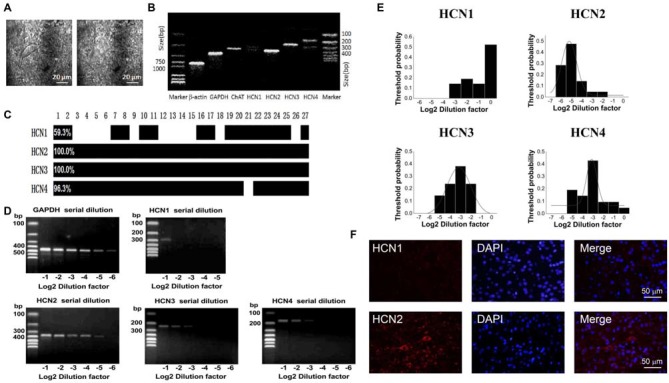
**HCN2 channel was dominantly expressed on ChIs. (A)** Schematic graph showed the procedure of cell aspiration, avoiding the nucleus. **(B)** PCR profile of a single ChAT-positive neuron had detectable level of all HCN channel subtypes, indicating the primer worked well. **(C)** Bar plot indicated the co-expression of HCN1–4 in ChAT-positive neurons detected by the multiple PCR. **(D)** Semi-quantitative scRT-PCR analysis of HCN channel expressed on ChIs. Note that HCN2 product was detectable with the sixth serial dilutions (2^−6^) of the template cellular DNA. **(E)** The histogram of threshold probability for ChIs expressing HCN channel subtypes. Subtypes except HCN1 showed a unimodal distribution. **(F)** Detection of HCN2 immunoreactivity in ChIs. Giant aspiny neuronal soma within dorsolateral striatum showed labeling of HCN2 subtype. HCN1 subtype staining was weak and individual neuron was not specifically visualized well.

In the striatum, the ChIs express HCN channels and characteristically have a pronounced hyperpolarization-activated sag potential (Kawaguchi, 1993; Bennett et al., 2000). To investigate the HCN channel identity expressed on ChIs, we carried out scRT-PCR experiment by sucking the cellular content into the recording pipette while avoiding its nucleus during the procedure. As shown in Figure 3A, the cell with large soma was aspirated, leaving a swollen nucleus in the original position. The cellular content of ChI was used for the conventional one-stage PCR amplification protocol. A representative PCR result was shown in Figure 3B. In this neuron, PCR products, separated by the electrophoresis in 2% agarose gels, stained with ethidium bromide and visualized by UV light, indicated that four isoforms of HCN channels were detected. Furthermore, HCN2 and HCN3 are more abundant compared with HCN4 and HCN1. The statistical results of 27 analyzed cells indicated that mRNAs of HCN2 and HCN3 were found in every neuron, whereas mRNAs of HCN1 and HCN4 were found in partial neurons with ratio of 11/27 and 26/27, respectively (Figure 3C).

The semi-quantitative RT-PCR experiment was performed in the modified two-stage amplification protocol to detect the relative abundance of HCN isoforms. Representative products amplified from serial diluted templates are shown in Figure 3D for each subtype. With Gaussian curve normalization, the HCN2 is highly translated into mRNA in ChIs. The threshold probability was best fit with a single Gaussian function (solid line) for all isoforms except HCN1. The detection threshold for HCN2 was 2^−5^, while it was 2^−3^ for HCN3 or HCN4 (Figure 3E). Considering the discrepancy between mRNA and protein expression, we further validate the protein level of HCN2 and HCN1 subtypes, which are the most and least abundant at mRNA level, through immnuohistochemical staining. As shown in Figure 3F, the use of subtype-specific antibody revealed a strong expression of HCN2 on large soma, which was identified as ChI morphologically, while HCN1 expression was relatively weak. These results confirmed that HCN2 is the main isoform expressed on ChIs.

### M Receptor-Dependent Decrease of Firing Activity

**Figure 4:**
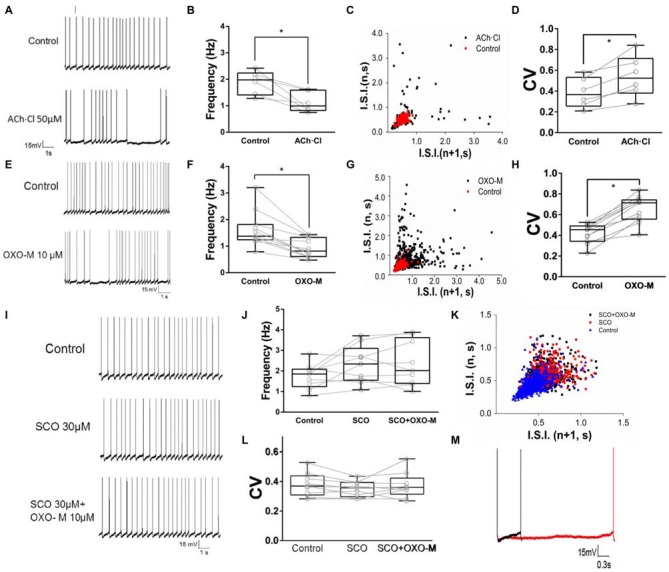
**M receptor activation reduces firing rate of ChIs. (A)** Example traces of whole-cell recordings of ChIs in control and after the application of 50 μM Acetylcholine (ACh)Cl. **(B)** ACh.Cl significantly decreased spontaneous firing frequency in control cells (*p < 0.05, paired student’s *t*-test, *n* = 6). **(C)** I.S.I. plot of the discharge of the neuron depicted in **(A)** (red, control; black, ACh.Cl). **(D)** ACh.Cl significantly increased CV of the I.S.I frequency (*p < 0.01, paired student’s *t*-test, *n* = 6). **(E)** Example traces of whole-cell recordings of ChIs in control and after the application of Oxotremorine (OXO-M) (10 μM). **(F)** OXO-M significantly decreased spontaneous firing frequency in control cells (*p < 0.01, paired student’s *t*-test, *n* = 11). **(G)** I.S.I. plot of the discharge of the neuron depicted in **(D)** (red, control; black, OXO-M). **(H)** OXO-M significantly increased the CV (*p < 0.001, paired student’s *t*-test, *n* = 11). **(I)** Example trace of whole-cell recording on ChIs in control and after the application of Scopolamine (SCO; 30 μM). SCO blocked the frequency reduction of OXO-M (10 μM). **(J)** SCO abolished the inhibition of OXO-M on spiking frequency while itself did not affected spontaneous firing in control cells (*p* > 0.05, *n* = 9, one-way analysis of variance (ANOVA)). **(K)** I.S.I. plot of the discharge of the neuron depicted in **(G)** (blue, control; red, SCO; black, SCO + OXO-M). **(L)** SCO significantly blocked the reduction effect of OXO-M on CV (*p* > 0.05, *n* = 9, one-way ANOVA analysis). **(M)** Amplification of the spike. Aligned action potential traces apparently showed a clear prolongation of the repolarization after the application of OXO-M (10 μM; black, control; red, OXO-M).

To observe the effect of M receptors on the firing activity of ChIs, ACh.Cl was use to activate M receptors. It was found that brief bath-application of ACh.Cl (50 μM) caused a powerful inhibition on the spontaneous firing activity of the recorded ChIs (Figure 4A). The spiking rate was slowed down (f_control_ = 1.88 ± 0.44, f_ACh±Cl_ = 1.13 ± 0.38, Figure 4B, *n* = 6, *p* < 0.05). In the presence of ACh, the firing pattern of ChIs was altered remarkably (Figure 4C), and CV was increased significantly from 0.38 ± 0.14 of the control group to 0.54 ± 0.20 (Figure 4D, *n* = 6, *p* < 0.01). In order to exclude the nicotinic (N) receptor-mediated effect, the M receptor agonist OXO-M (10 μM) was applied. OXO-M (10 μM) displayed a similar effect as ACh.Cl (Figure 4E). The spiking pattern was changed (Figures 4E,G) and the spiking rate was slowed down (f_control_ = 1.62 ± 0.67, f_OXO-M_ = 0.93 ± 0.35, Figure 4F, *n* = 11, *p* < 0.01). The median CV was increased from 0.41 ± 0.09 of control to 0.65 ± 0.13 in the presence of OXO-M (Figures 4G,H, *n* = 11, *p* < 0.001). Moreover, the application of OXO-M profoundly increased the duration of hyperpolarization controlled by HCN channel (Figure 4M). Application of SCO (30 μM), a specific antagonist of M receptors, did not alter the spiking frequency of ChIs (f_control_ = 1.75 ± 0.61, f_SCO_ = 2.31 ± 0.91, Figures 4I,J, *n* = 9, *p* > 0.05). However, the inhibitory effect of OXO-M was abolished in the presence of SCO (f_control_ = 1.75 ± 0.61, f_SCO+OXO−M_ = 2.35 ± 1.10, Figures 4I,J, *n* = 9, *p* > 0.05). The spiking pattern of ChIs was not changed (Figure 4K) and the median CV was not altered with statistical significance when both SCO and OXO-M was applied continuously (CV_control_ = 0.38 ± 0.08, CV_SCO_ = 0.35 ± 0.05, CV_SCO+OXO−M_ = 0.37 ± 0.09, Figure 4L, *n* = 9, *p* > 0.05). The results suggested that M receptors were involved in the negative modulation on the firing activity of ChIs.

### Muscarinic Modulation of I_h_in ChIs

**Figure 5:**
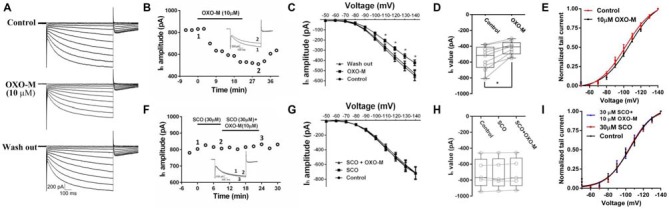
**Inhibition of OXO-M on I_h_ through M receptor activation. (A)** A representative trace of reversible inhibition effect of OXO-M on I_h_. **(B)** Schematic scatter plot showed the time-related effect of OXO-M (10 μM) on I_h_. **(C)** I_h_ activation I-V response curve before and after addition of OXO-M. **(D)** Schematic box-whiskers figure indicated the maximum current amplitude was inhibited by OXO-M at the −140 mV voltage stimulus. **(E)** Application of OXO-M caused a shift of voltage-dependent activation of I_h_ in the hyperpolarizing direction. **(F)** M receptor antagonist SCO (30 μM) had no discernable effect on I_h_ and blocked the inhibition of OXO-M (10 μM). **(G)** I–V response curve indicated inhibitory effect of OXO-M on I_h_ was blocked by M receptor antagonist. **(H)** Box plot summary of the change in I_h_ value after blocked by SCO. OXO-M can’t significantly decrease the I_h_ in the presence of SCO even with the maximum voltage stimulus (*P* > 0.05, *n* = 5, one-way ANOVA analysis). **(I)** Application of SCO blocked the shift of voltage-dependent activation of I_h_, and SCO failed shift the response in the hyperpolarizing direction.

Previous evidence indicated that M receptor could regulate many ionic channels involved in autonomous spiking (Yan and Surmeier, 1996; Calabresi et al., 1998; Goldberg and Wilson, 2005; Ding et al., 2006). In the present study, we found that OXO-M (10 μM) inhibited I_h_ apparently (Figures 5A–C). A representative curve illustrated in Figure 5B indicated that the maximum inhibitory effect appeared after OXO-M was added for ~20 min. When the stimulus voltage was hyperpolarized, OXO-M exhibited significant inhibition on I_h_ current (Figure 5C). As shown in Figure 5D, the maximum I_h_ current evoked at −140 mV showed a statistical significant reduction after the addition of OXO-M. The amplitude of I_h_ was decreased by 21.1% ± 8.5% (Figure 5D, *n* = 9, *p* < 0.01). The peak amplitudes of tail currents were normalized and fitted by Boltzmann function to determine the voltage-dependent activation (Deng et al., 2007). As shown in Figure 5E, OXO-M caused a hyperpolarizing shift in the activation of I_h_. The half-activation voltage (V_1/2_) was altered to −107.4 ± 2.25 mV from −102.4 ± 2.11 mV in the presence of OXO-M (*n* = 9, *p* < 0.01). SCO, 30 μM, was added into the buffer to affirm that the inhibition was mediated by the direct interaction of OXO-M on M receptors. As shown in Figures 5F,G, the time- and voltage-related inhibitory effect of OXO-M was abolished by SCO. At the maximum voltage level, OXO-M did not display statistical significant depression of I_h_ in the presence of SCO (99.2% ± 0.03% of control, Figure 5H, *n* = 5, *p* > 0.05). The presence of SCO occluded the alteration of V_1/2_ in OXO-M, while itself did not altered the V_1/2_ (V_1/2_ in control, −105.7 ± 2.23 mV; V_1/2_ in SCO, −104.0 ± 1.76 mV; V_1/2_ in SCO + OXO-M, −104.5 ± 2.50 mV; Figure 5I, *n* = 5, *p* > 0.05), which demonstrated that M receptor was involved in the I_h_ reduction.

### M Receptors Expression on ChIs

**Figure 6:**
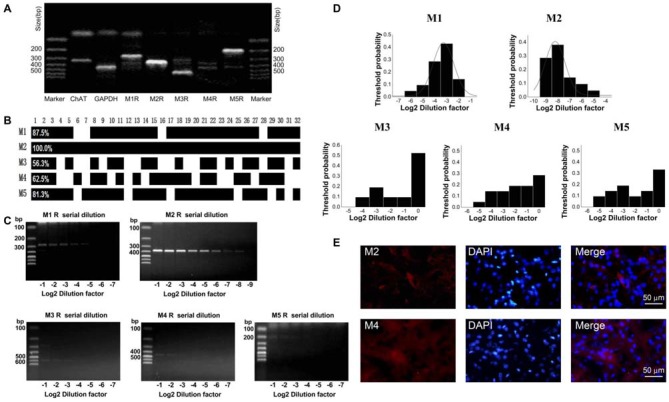
**M2 receptor was dominantly expressed on ChIs. (A)** PCR profile of a single ChAT-positive neuron that has detectable levels of all M receptor subtypes indicated the primer worked well. **(B)** Bar plot showed the co-expression of M1-M5 in ChAT-positive neurons. **(C)** Semi-quantitative scRT-PCR analysis of M receptor expressed on ChIs. Note that M2 receptor PCR amplicons were detectable with the eighth dilutions (2^−8^) of the template cellular DNA. **(D)** The histogram of threshold probability for ChIs expressing M receptor. M1 and M2 showed a unimodal distribution.** (E)** Giant aspiny neuronal soma within dorsolateral striatum showed strong immunoreactivity to M2 receptor, while M4 receptor immunostaining was not well visualized.

To determine which subtype of M receptors plays a dominant role in mediating the inhibitory effect of OXO-M, we conducted a thoroughly analysis of M receptor subtypes expressed on ChIs. Similar to the analysis of HCN subtypes, we analyzed 32 single neurons utilizing scRT-PCR technique. mRNAs of five M receptor subtypes were detected using the conventional one-stage amplification protocol (Figure 6A). The lanes of PCR products stained with ethidium bromide in 2% agarose gels indicated that M2 was the most abundant one (Figure 6A). As shown in Figure 6B, M2 receptor mRNA was expressed in all detected neurons, whereas only a small subset of neurons (20/32) had detectable levels of M4 mRNA. Meanwhile, M1-like receptors (M1, 3, 5), which were mainly expressed on projection neurons, were also detected on ChIs in a ratio of 28/32, 18/32, 26/32 respectively.

We, then performed the semi-quantitative RT-PCR experiment to discover the relative abundance of M receptors. The representative single-cell serial dilution gels were shown for each subtype in Figure 6C. M2 exhibited stronger signal than other four isoforms. The threshold probability of M1 and M2 were fit well with a single Gaussian function (solid line). As Gaussian curve normalization indicated, the detection threshold was 2^−8^ for M2, while 2^−3^ for M1, respectively (Figure 6D). The other three, M3, M4, and M5, were in low abundance that was barely above the detection level and Gaussian function could not provide an estimate of central tendency. We selected M2 and M4 to perform histochemical staining. The high expression of M2 receptor in ChIs, identified as ChI morphologically, was demonstrated by highlighted fluorescence staining. While with M4 receptor labeling, there was intense and heterogeneous staining of the striatal neuropil but individual neuron was not well visualized (Figure 6E). These results confirmed that M2 was the dominant isoform expressed on ChIs.

### Gi-Protein Dependence and Interaction with M2 Receptor

**Figure 7:**
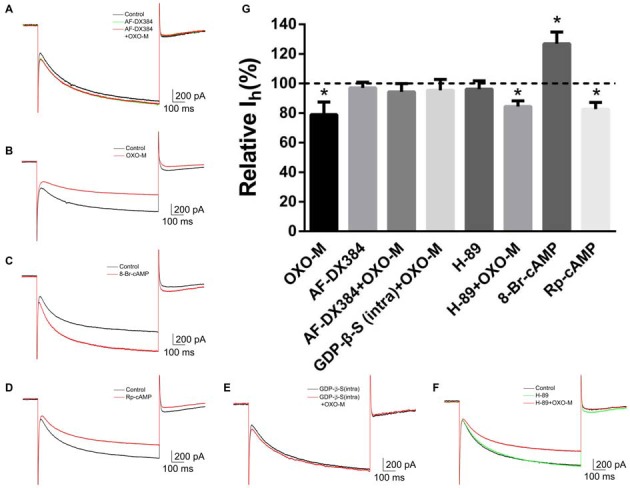
**The inhibitory effect of OXO-M on I_h_ was mediated by M2 receptor activating Gi protein coupled signaling pathway. (A–F)** schematic illustrations of I_h_ trace at the −140 mV hyperpolarizing voltage before and after application of each drug. **(G)** Summarized bar graph of drugs’ effect. Bath application of AF-DX384 (1 μM) did not alter I_h_, but fully blocked OXO-M induced inhibition on I_h_. The effect of OXO-M was fully abolished by GDP-β-S (0.5 μM), while was not affected by H-89 (10 μM). The I_h_ was enhanced in the presence of 8-Br-cAMP (100 μM) and reduced by Rp-cAMP (50 μM). *p < 0.01 compared with the normalized control.

To explore the mechanism underlying OXO-M inhibition on I_h_, we first observed the effect of M2 receptor which is dominantly expressed on ChIs. It was found that AF-DX384, a M2-like receptor antagonist, did not show any visible effect on I_h_ (97.0% ± 3.8% of control, Figures 7A,G, *n* = 8, *p* > 0.05). However, the inhibitory effect of OXO-M (10 μM) on I_h_ was abolished after application of AF-DX384 (1 μM). The average relative I_h_ was changed from 78.9% ± 8.5% (Figures 7B,G, *n* = 9) in the OXO-M alone to 94.3% ± 5.5% in the presence of both OXO-M and AF-DX384 (Figures 7A,G, *n* = 6).

To investigate the post-receptor signaling transduction, 8-Br-cAMP (100 μM), a membrane-permeable cAMP analog, was used as Deng et al. (2007) described. The amplitude of I_h_ current was raised to 126.8% ± 8.0% (*n* = 4, *p* < 0.01) in the presence of 8-Br-cAMP (Figures 7C,G). On the other hand, application of Rp-cAMP (50 μM), a specific inhibitor of the cAMP signaling pathway, resulted in a significant reduction of I_h_ (82.6% ± 4.5% of control, Figures 7D,G, *n* = 4, *p* < 0.01). GDP-β-S, an unhydrolyzable GDP analog, competes with endogenous GTP for the nucleotide binding site on G-proteins, locking G-proteins in an inactive state. When pipettes were loaded with GDP-β-S (0.5 μM), the response of I_h_ to OXO-M was prevented (95.4% ± 7.3% of control, Figures 7E,G, *n* = 7, *p* > 0.05). H-89 inhibits cAMP-dependent protein kinase selectively and potently. Our results revealed that H-89 (10 μM) did not present notable inhibition on I_h_ (96.2% ± 5.5% of control, Figures 7F,G, *n* = 7, *p* > 0.05) and co-application of H-89 (10 μM) with OXO-M (10 μM) did not hinder the inhibitory effect of OXO-M either (84.4% ± 3.8% of control, Figures 7F,G, *n* = 11, *p* < 0.01). These data support the conclusion that muscarinic inhibition of I_h_ is mediated through a PKA-independent cAMP pathway.

## Discussion

Striatal ChIs were first identified in Kölliker (1896). Extracellular recording in the striatum of awake and normal behaving primates revealed the presence of tonically active neurons that possess particularly broad action potentials (Aosaki et al., 1995). In the past decade, studies of the ChIs have further expanded the understandings of striatal regulation and innervation in the signal input of basal ganglia circuit (Cragg, 2006; Ding et al., 2006; Pisani et al., 2007; Exley and Cragg, 2008). ChIs in the striatum have been considered a major modulator for the duration, strength, and spatial pattern of striatal MSNs output by releasing neurotransmitter ACh, which activates the postsynaptic M receptors.

As M receptors are also expressed on ChIs, we speculate that ACh, released by ChIs themselves, may affect their own excitability by activating these autoreceptors. To test this hypothesis, we identified the subtypes of M receptors expressed on ChIs and observed the effect of ACh on their firing activity. Because HCN channels are considered to contribute regulatory effects on ChIs’ excitability (Bennett et al., 2000; Wilson, 2005), we also identified the subtypes of HCN channels expressed on these neurons, and then explored the effect of M receptor agonists on I_h_.

### Subtypes of M Receptors and HCN Channels Expressed on ChIs

Comprising only 1–3% of all striatal neurons, ChIs have widespread and rich connections within the striatum (Woolf and Butcher, 1981; Nastuk and Graybiel, 1985). Combined morphological, histochemical, and electrophysiological features together, we confirmed the recording neurons were ChIs (Kemp and Powell, 1971; Wilson et al., 1990; Kawaguchi, 1993; Bennett et al., 2000; Wilson, 2005). We, found most of recorded neurons showed a spontaneous firing without artificial stimulation. The ChIs characteristically elicited a large amplitude and long duration AHP after repetitive firings by injected depolarizing current and displayed a pronounced hyperpolarization-activated sag potential. These features coincide with that previously reported (Calabresi et al., 1998; Bennett and Wilson, 1999; Ponterio et al., 2013).

Extensive M2 and M1 mRNAs in ChIs have been found (Bernard et al., 1992). M2-class receptors are recognized as autoreceptors, while M4 receptor is mainly expressed by a subpopulation of striatal projection neurons (Hersch et al., 1994; Bernard et al., 1999). The expression of M receptors on cultured striatal ChIs has been reported previously (Yan and Surmeier, 1996). In the present study, we analyzed the M receptor mRNA of ChIs in brain slice which is different from the report of Yan and Surmeier (1996). We, found that all five subtypes of M receptors were transcribed on ChIs. In 32 ChAT-positive neurons, the percentage of cells transcribing mRNA of M1 to M5 is 87.5%, 100.0%, 56.3%, 62.5%, and 81.3%, respectively. These results agree with the report of Yan and Surmeier (1996) that the M2-like receptors are highly transcribed while M1-like receptors are relative low in ChIs. Combining with the immunohistochemical staining results, M2 subtype is found in every ChI. Our findings are consistent with previous reports that M2 is the main subtype on ChIs (Weiner et al., 1990; Levey et al., 1991).

Earlier studies reported that ChIs expressed HCN channel subtypes of HCN2, HCN3, and HCN4 (Santoro et al., 2000; Notomi and Shigemoto, 2004), but not HCN1 (Santoro et al., 1998; Chen et al., 2001). Indeed, our data revealed that HCN2, HCN3, and HCN4 are transcribed in nearly all studied neurons by using multiplex PCR. However, HCN1 mRNA was also found on a portion of ChIs (16/27). The presence of HCN1 mRNA may due to the increments of amplified cycling number. The relative contents of all subtypes in our work are resembled with the form mentioned earlier studies. Consistent with scRT-PCR results, immunohistochemical staining directly indicated HCN2 channel fluorescence is strong and HCN1 staining is weak (Figure 4E). Among the four subtypes of HCN channels, HCN1 is activated fastest and weakly sensitive to cAMP. HCN2 has a slow activating kinetics and highest sensitivity to intracellular cAMP (Chen et al., 2001; Wang et al., 2002; Ulens and Siegelbaum, 2003). The I_h_ currents we recorded in ChIs display a high sensitivity to cAMP. Intracellular application of cAMP analog (8-Br-cAMP, 100 μM) enhances the current remarkably (Figure 7). These data are consistent with the notion that I_h_ recorded from a whole cell of striatal ChIs presents a HCN2-like characteristic.

### Muscarinic Modulation of I_h_ in Cholinergic Interneurons

ChIs’ spiking is regulated through many ionotropic channels and G-protein coupled receptors (Yan et al., 1997; Calabresi et al., 2000; Zhou et al., 2002; Maurice et al., 2004; Wilson, 2005), and I_h_ plays a vital role in determining the firing rate of ChIs (Bennett and Wilson, 1999; Bennett et al., 2000; Maurice et al., 2004; Wilson, 2005). Accumulating evidence indicates that the activity of the I_h_ channel is regulated by a variety of neuromodulators (Pape, 1996; Frère et al., 2004). The dopaminergic modulation of I_h_ depends on receptor subtypes (i.e., D_1_- or D_2_- like receptors). Previous studies have shown that dopamine inhibits I_h_, mediated through D2-like receptors in stratum (Deng et al., 2007) and ventral tegmental neurons (Jiang et al., 1993), whereas enhances I_h_ via a synergistic activation of D1- and D2- like receptors in neocortex layer I interneurons (Wu and Hablitz, 2005). Activation of β1 noradrenergic receptor or 5-HT receptor enhances I_h_ through a cAMP-dependent mechanism (Bobker and Williams, 1989; Pisani et al., 2003; Blomeley and Bracci, 2005; Bonsi et al., 2007; Hawkins et al., 2015). The regulation of M receptors on the spike of ChIs was also reported previously (Yan and Surmeier, 1996; Calabresi et al., 1998; Ding et al., 2006). Modulations on Na^+^ channel (Maurice et al., 2004), Ca^2+^ channel (Yan and Surmeier, 1996; Ding et al., 2006, 2010), and K^+^ channel (Song et al., 1998; Goldberg and Wilson, 2005) could contribute to the M receptor regulating effect. Though the interaction between M receptors and HCN channels have been hypothesized for a long time, whether I_h_ involved directly in the muscarinic regulation on ChIs’ spiking remained to be demonstrated.

As reported, ChIs are the main source of ACh in the striatum and produce a wide innervation over the entire striatal complex (Phelps et al., 1985; Phelps and Vaughn, 1986; Goldberg and Reynolds, 2011). Our present results demonstrate that ChIs expressed M receptor and HCN channel abundantly, and their spontaneous firing can be modulated by application of M receptor agonist or HCN channel blocker. OXO-M inhibited spiking and prolonged the AHP repolarizing time, and these correlated with a decrease in I_h_ current. The effect of OXO-M on I_h_ is blocked by application of antagonist SCO and M2-like receptor selective antagonist AF-DX384, which suggests that the activation of M2-like receptor is the prerequisite for OXO-M inhibition. Therefore, we concluded that M2-like receptors are playing a critical role in ACh-mediated effect. However, the role of which M2-like receptor, M2 or M4, plays in the ACh autoregulation needs to be demonstrated further with specific muscarinic subtype antagonists.

In our study, SCO and AF-DX384 failed to enhance I_h_, while SCO slightly improved the spiking frequency of ChIs without statistical significance, as described above. These inferred that the auto-regulation mediated by presynaptic M receptor on ChIs was attenuated as previously reported (Calabresi et al., 1998). As described, the majority of ChIs are spontaneous firing in brain slices (Bennett and Wilson, 1999), there should be a background regulation of intrinsic ACh. We speculated the attenuation may induced by the following factors. Firstly, the termination of intact thalamic projection in coronal slices may attribute to the attenuation of heterosynaptic contacts and the destruction of local cholinergic circuit in striatum may result in the attenuation of hotorosynaptic contacts (Phelps et al., 1985; Hersch et al., 1994). Secondly, the whole-cell recording with low-resistance pipettes can compromise I_h_ likely via dilution of intracellular cAMP. In order to avoid these artificial interferences and observe the auto-inhibition directly and persuasively in electrophysiology, perhaps a cell-attached recording technique would be better suited to identifying the autoregulatory function. Otherwise, it would need *in vivo* patch clamp recording or/and extrinsic stimulation.

We find that both HCN channel blocker ZD7288 (Figure 2) and M receptor agonist ACh and OXO-M (Figure 4) could depress the firing activity of ChIs. However, the mechanism between the regulation of HCN channel and M receptor is unclear. The high expression of cAMP sensitive HCN channels provides a common pathway for G protein coupled receptors to regulate the ChIs activity. As previously reported, cAMP could directly combine with HCN channels on the site of CNBD domain (Pape, 1996; Wang et al., 2001; Young and Krougliak, 2004), leading to enhancement of I_h_ current. The reported effects of dopamine (Deng et al., 2007) and noradrenaline (Pape and McCormick, 1989; McCormick and Pape, 1990) on I_h_ current come to depend on the regulation of intracellular cAMP levels. These regulations are of great importance for ChIs’ spiking activity that will finally project to the output neurons of striatum (Doležal and Tuček, 1998).

As shown in Figure 7, the inhibitory effect of OXO-M is also abolished when GDP-β-S is used to fix G-proteins in an inactive state, but not while H-89 is used to inhibit cAMP-dependent protein kinase, and the effect were also mimicked in the presence of cAMP analog as reported (Deng et al., 2007). These data demonstrate that the G_i/o_/cAMP signaling pathway is involved in muscarinic modulation on the excitability of ChIs, but not cAMP-dependent PKA signaling cascade. These results are consistent with the direct regulation of intracellular cAMP on HCN channel (Pedarzani and Storm, 1995; Robinson and Siegelbaum, 2003). In addition to the physiological implications for neuronal rhythmic activity, our results also suggest a novel mechanism for dynamic signaling through second messenger. It is possible that cholinergic terminals involved in this muscarinic inhibitory circuit in the striatum are associated with different behavioral contexts.

## Conclusion

Our data reveal that all known subtypes of M receptors and HCN channels are transcribed in striatal ChIs. Among them, M2 and HCN2 are the most abundant ones. The spontaneous spiking of ChIs could be inhibited by extrinsic application of M receptor agonists or HCN channel blockers. Muscarinic agonists exert the inhibition on the excitability of ChIs probably by activating M2-like receptors, reducing intracellular cAMP and finally depressing HCN2 channels. These results may imply that ChIs possibly receive a negative feedback modulation by ACh *in vivo*.

## Author Contributions

Study concept design: ZZ, JZ, LW, and XW. Collection of data: ZZ and KZ. Analysis and interpretation of data: ZZ, JZ. Drafting of the manuscript: ZZ, KZ. Critical revision of the manuscript: JZ. Study supervision: XL, HY, XM, and SZ. All authors approved the final version of the manuscript. All experiments were performed in State Key Laboratory of Toxicology and Medical Countermeasures in China.

## Conflict of Interest Statement

The authors declare that the research was conducted in the absence of any commercial or financial relationships that could be construed as a potential conflict of interest.
